# Ameliorating Effect of Klotho Protein on Rat Heart during I/R Injury

**DOI:** 10.1155/2020/6427284

**Published:** 2020-10-17

**Authors:** Agnieszka Olejnik, Anna Krzywonos-Zawadzka, Marta Banaszkiewicz, Iwona Bil-Lula

**Affiliations:** Division of Clinical Chemistry and Laboratory Hematology, Department of Medical Laboratory Diagnostics, Faculty of Pharmacy, Wroclaw Medical University, Borowska 211A St., 50-556 Wroclaw, Poland

## Abstract

An essential procedure for the treatment of myocardial infarction is restoration of blood flow in the obstructed infarct artery, which may cause ischaemia/reperfusion (I/R) injury. Heart I/R injury manifests in oxidative stress, metabolic and morphological disorders, or cardiac contractile dysfunction. Klotho protein was found to be produced in the heart tissue and participate in antioxidation or ion homeostasis. The aim of this study was to examine an influence of Klotho protein on the heart subjected to I/R injury. Wistar rats served as a surrogate heart model *ex vivo*. Rat hearts perfused using the Langendorff method were subjected to global no-flow ischaemia, and isolated rat cardiomyocytes underwent chemical I/R *in vitro*, with or without recombinant Klotho protein administration. Haemodynamic parameters of heart function, cell contractility, markers of I/R injury and oxidative stress, and the level of contractile proteins such as myosin light chain 1 (MLC1) and troponin I (TnI) were measured. The treatment of hearts subjected to I/R injury with Klotho protein resulted in a recovery of heart mechanical function and ameliorated myocyte contractility. This improvement was associated with decreased tissue injury, enhanced antioxidant capacity, and reduced release of MLC1 and TnI. The present research showed the contribution of Klotho to cardioprevention during I/R. Thus, Klotho protein may support the protection from I/R injury and prevention of contractile dysfunction in the rat heart.

## 1. Introduction

Restoration of the flow in the obstructed infarct artery is pivotal for the treatment of myocardial infarction. However, revascularization and restoration of the blood flow inflict myocardial ischaemia/reperfusion (I/R) injury. I/R injury can be irreversible, and it manifests itself in increased infarct size and microvascular dysfunctions [1, 2]. It is known that I/R leads to oxidative stress and the subsequent cascade of pathophysiological events in the heart. The important factors contributing to the pathogenesis of I/R injury are the degradation of heart contractile proteins by proteolytic enzymes and necrotic cell death. The proteolytic enzymes like matrix metalloproteinases (MMPs) degrade cardiac troponin, titin, or myosin light chains (MLCs). As a result, numerous metabolic, morphological, and contractile disorders in the myocardium are observed [3]. The approach of regulating oxidative stress in the heart may support the reduction of maladaptive response established during I/R injury. Therefore, searching for new factors contributing to the prevention and treatment of myocardial injury following I/R is needed.

Klotho is an antiaging protein that is bounded to the cell membrane or released into extracellular space and then found in the blood, urine, or cerebrospinal fluid. The main function of Klotho is the regulation of fibroblast growth factor (FGF) signalling and ion homeostasis. There is a lot of evidence for the protective role of Klotho in the kidney, the main organ that produces Klotho protein. The expression of Klotho was observed also in the pituitary gland, placenta, skeletal muscle, urinary bladder, aorta, pancreas, testis, ovary, colon, or thyroid gland. *β*Klotho, other Klotho family member, is expressed in the adipose tissues, liver, pancreas, and some part of the brain [4–8]. An intracellular Klotho form in the cytoplasm of mouse kidney and human parathyroid gland cells was also found. Importantly, intracellular Klotho served as an endogenous anti-inflammatory and antiaging factor [6, 7]. It has been shown that Klotho was involved in the kidney or brain protection by regulation of oxidative stress, inflammation, and apoptosis [5, 9–11]. Moreover, Klotho deficiency correlated with the occurrence and development of atherosclerosis, coronary artery disease, myocardial infarction, and left ventricular hypertrophy [7]. We have reported the expression of Klotho in the cardiomyocytes recently [12]. However, a cell line model does not include the effect of the intercellular interactions and signalling between the cardiomyocytes and other types of cells in the cardiac tissue. For this reason, we decided to examine Klotho influence at the tissue level using Wistar rat hearts. We propose that Klotho may contribute to the protection of the heart against I/R injury in the current research.

This study was aimed at investigating the influence of Klotho protein on the rat hearts subjected to I/R injury.

## 2. Materials and Methods

### 2.1. Experimental Animals

Adult male Wistar rats weighing 200-350 grams were obtained from Mossakowski Medical Research Center, Polish Academy of Sciences, Warsaw, Poland. The animals were housed in cages (two rats/cage) and kept at controlled temperature (22 ± 2°C), humidity (55 ± 5%), and light/dark (12/12 hours) cycle. An *ad libitum* access to a diet of standard laboratory chow and water was provided. All experimental procedures in the animals were performed following the published Guide of the Polish Ministry of Science and Higher Education for the Care and Use of Experimental Animals. This investigation was approved by the Ethics Committee for Experiments on Animals at the Ludwik Hirszfeld Institute of Immunology and Experimental Therapy Polish Academy of Sciences, Wroclaw, Poland (Resolution 002/2020 of 15th January 2020).

### 2.2. Isolated Rat Hearts Perfused with the Langendorff Method

Rats were desensitized with buprenorphine (0.05 mg/kg, i.p.) and anesthetized with sodium pentobarbital (0.5 ml/kg i.p.), and the hearts were rapidly excised from animals. The spontaneously beating hearts were then rinsed by immersion in ice-cold Krebs-Henseleit Buffer (118 mmol/L NaCl, 4.7 mmol/L KCl, 1.2 mmol/L KH_2_PO_4_, 1.2 mmol/L MgSO_4_, 3.0 mmol/L CaCl_2_, 25 mmol/L NaHCO_3_, 11 mmol/L glucose, and 0.5 mmol/L EDTA, pH 7.4) and cannulated by the aorta on a Langendorff system (EMKA Technologies, Paris, France). The above procedure was completed within 30 sec. Then, hearts were perfused at constant pressure (60 mmHg) with Krebs-Henseleit Buffer (pH 7.4, 37°C) and gassed continuously (5% CO_2_/95% O_2)_. A latex balloon was filled with water, and then, it was connected to a pressure transducer (EMKA Technologies, Paris, France). Then, the balloon was introduced into the left ventricle via a mistrial valve. The volume of the balloon was adjusted to achieve a stable left ventricular end-diastolic pressure of 8-10 mmHg during stabilization and reperfusion. The following haemodynamic parameters using an EMKA recording system with IOX2 software (EMKA Technologies, Paris, France) were monitored: coronary flow (CF), heart rate (HR), left ventricular developed pressure (LVDP), left ventricular end-diastolic pressure (PED), and intraventricular pressure (dP/dt). Hearts that showed CF > 28 mL/min or <10 mL/min were excluded from the study.

### 2.3. Global Ischaemia/Reperfusion Injury of Isolated Rat Hearts

**Figure 1:**
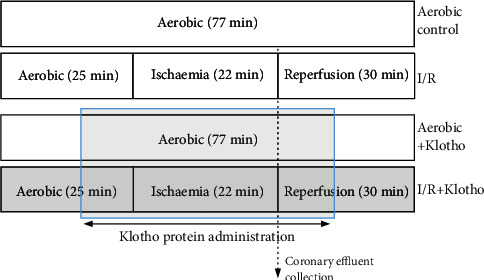
Experimental protocol for I/R injury of isolated rat hearts, with and without Klotho administration. Klotho protein was added into the perfusion buffer and administered to isolated hearts during the last 10 min of aerobic stabilization (prior to the global ischaemia) and within the first 10 min of reperfusion after global ischaemia. I/R: ischaemia/reperfusion.

Rat hearts were distributed equally and randomly into 4 groups: aerobic control without Klotho (*n* = 12), aerobic control with Klotho (*n* = 3), acute myocardial I/R injury without (*n* = 14), and with Klotho (*n* = 7). The scheme of the experimental protocol of heart ischaemia/reperfusion injury is shown in Figure 1. No injury of isolated hearts prior to global ischaemia was established in the preliminary experiment by measurement of the activity of LDH released from hearts (a marker of cell injury). There was no difference between groups in LDH activity in coronary effluents collected at 25 minutes of the experiment (data not shown). The isolated hearts from I/R groups underwent 25 min of aerobic stabilization, 22 min of global no-flow ischaemia (by a cessation of the buffer flow), and 30 min of reperfusion (aerobic conditions) [13], in the presence or absence of Klotho protein. The hearts from aerobic groups were perfused aerobically for 77 min, with or without Klotho protein administration. Recombinant Rat *α*Klotho protein (Cloud-Clone Corp., RPH757Ra01) was diluted to a final concentration of 0.5 ng/mL with Krebs-Henseleit Buffer, immediately before administration. The optimal concentration of Klotho protein was determined experimentally. Klotho was administered with the perfusion buffer into the hearts during the last 10 min of aerobic stabilization and in the first 10 min of reperfusion (after global ischaemia) [13, 14] (Figure 1). To determine cardiac mechanical function, the recovery of rate pressure product (RPP) was expressed as the product of HR and LVPD and evaluated at 25 min of the experiment (the end of aerobic perfusion) and at 77 min (the end of reperfusion) [13, 14]. After the experimental protocol, isolated hearts were immediately immersed in liquid nitrogen and stored at −80°C for further investigations. 15 mL of coronary effluents was collected at the beginning of reperfusion (47 min of the experiment) (Figure 1). Then, coronary effluents were concentrated (1 mL final volume) using Amicon Ultra-15 Centrifugal Filter Units with Ultracel-10 membrane (EMD Millipore, USA), aliquoted, and frozen at -80°C for further biochemical analysis.

### 2.4. Heart Perfusion and Isolation of Ventricular Rat Cardiomyocytes

Rats were treated with buprenorphine (0.05 mg/kg, i.p.) and anaesthetized with sodium pentobarbital (0.5 mL/kg i.p.). The hearts were rapidly excised from animals and rinsed immediately after removal by immersion in ice-cold Myocyte Isolation Buffer (MIB) containing 120 nmol/L NaCl, 5 mmol/L KCl, 2 mmol/L NaAc, 2 mmol/L MgCl_2_, 1 mmol/L Na_2_HPO_4_, 20 mmol/L NaHCO_3_, 5 mmol/L glucose, 9 mmol/L taurine, and 10 mmol/L CaCl_2_ at pH 7.4. The spontaneously beating hearts were suspended on a blunt-end needle by the aorta in a Langendorff system and perfused with MIB containing 1 mmol/L CaCl_2_ at 37°C for 5 min. To induce the loss of contractility of cardiomyocytes, the buffer was replaced with MIB containing 5 *μ*mol/L CaCl_2_. Then, the right ventricle was excised after mild swelling of the myocardium in HEPES buffer (120 mM NaCl 140, 5 mM KCl, 2 mM MgCl_2_, 5 mM glucose, 9 mM taurine, and 5 mM HEPES) containing 40 *μ*mol/L CaCl_2_, collagenase, and protease. The ventricle was rinsed with HEPES buffer containing 100 *μ*mol/L CaCl_2_ and 150 mg bovine serum albumin and minced into small pieces in the digestion solution (HEPES buffer containing 100 *μ*mol/L CaCl_2_, 150 mg BSA, 15 mg collagenase, and 1 mg protease). The minced tissue was repeatedly digested six times for 20 and 10 min in a water bath (37°C). The 3rd-6th fraction was used for further experiments.

### 2.5. Chemical Ischaemia/Reperfusion of Isolated Ventricular Rat Cardiomyocytes

Isolated rat cardiomyocytes underwent 15 min of aerobic stabilization in HEPES buffer containing 100 *μ*mol/L CaCl_2_ and 150 mg BSA. Then, the ischaemia was induced by covering the cell pellet with HEPES buffer containing 4 mmol/L 2-deoxyglucose (to inhibit glycolysis) and 40 mmol/L sodium cyanide (an inhibitor of cellular respiration) for 3 min (I/R groups). The optimal duration of ischaemia was established in our previous studies [13, 15]. Then, the reperfusion was conducted by removing the buffer containing sodium cyanide by centrifugation (1 min, 1500 × g) and incubation of cell pellet for 20 min in a fresh portion of HEPES buffer (100 *μ*mol/L CaCl_2_, 150 mg BSA). In the I/R+Klotho group, cells were incubated in the appropriate buffers with Recombinant Rat *α*Klotho protein (Cloud-Clone Corp., RPH757Ra01) during aerobic stabilization, ischaemia, and reperfusion (whole experimental protocol of I/R). The following concentrations of Klotho in the buffers were tested: 100 ng/mL, 5 ng/mL, and 0.5 ng/mL (final concentration). Cells from the aerobic control group were maintained in aerobic conditions and kept exposed to atmospheric air for 38 min in HEPES buffer (100 *μ*mol/L CaCl_2_, 150 mg BSA). For contractility measurement, cells were centrifuged at 1500 × g for 5 min and suspended in HEPES buffer (100 *μ*mol/L CaCl_2_, 150 mg BSA).

### 2.6. Measurement of Ventricular Rat Cardiomyocyte Contractility

After the protocol of I/R injury, a 100 *μ*L aliquot of cell suspension was placed in the rapid change stimulation chamber of the IonOptix Contractility System (IonOptix, Milton, MA, USA). The cardiomyocytes were stabilized for 3 min and perfused with oxygenated HEPES buffer containing 2 mmol/L CaCl_2_ (4 mL/min) at 37°C. The contractility was measured by continuously pacing the cells with 1 Hz and 5 V (MyoPacer, IonOptix) and expressed as a percent of peak shortening in comparison to the length of the diastolic cell. At least an average of six cells per sample and six samples per experimental condition was evaluated.

### 2.7. Preparation of Heart Homogenates

Frozen hearts were crushed using a mortar and pestle in liquid nitrogen. Then, heart tissue was homogenized mechanically in ice-cold homogenization buffer (50 mmol/L Tris-HCl (pH 7.4), 3.1 mmol/L sucrose, 1 mmol/L dithiothreitol, 10 mg/mL, leupeptin, 10 mg/mL soybean trypsin inhibitor, 2 mg/mL aprotinin, and 0.1% Triton X-100). The homogenate was centrifuged (10 000 × g at 4°C for 15 min), and the supernatant was collected and stored at −80°C for further biochemical experiments.

### 2.8. Assessment of LDH Activity

Lactate dehydrogenase (LDH) activity served as a marker of heart injury. The activity of LDH was assessed with the Lactate Dehydrogenase Activity Assay Kit (Sigma-Aldrich) according to the manufacturer's instruction. LDH is a stable cytosolic enzyme that is released into the extracellular space upon cell membrane damage or permeability. Briefly, LDH interconverts pyruvate and lactate with the reduction of NAD to NADH, which is detected with a colorimetric assay at 450 nm. LDH activity was determined in coronary effluents and normalized to CF.

### 2.9. Assessment of Cytotoxicity Level in Rat Hearts

To evaluate an influence of I/R injury and Klotho on isolated rat hearts, the cytotoxicity level using the CytoTox-Glo™ Cytotoxicity Assay (Promega, Madison, WI, USA) according to the manufacturer's instruction was assessed. The assay measures the relative number of dead cells based on the extracellular activity of a distinct intracellular protease (dead-cell protease). Dead-cell protease is released from membrane-compromised or damaged cells. Briefly, to measure extracellular dead-cell protease activity, a luminogenic cell-impermeant peptide substrate (alanyl-alanyl-phenylalanyl-aminoluciferin; AAF-aminoluciferin) is used. Dead-cell protease cleaves the luminogenic AAF-aminoluciferin substrate. Then, the Ultra-Glo™ Recombinant Luciferase is used for the measurement of luminescence generated by liberated aminoluciferin product. The intensity of luminescence is proportional to the percentage of cells undergoing cytotoxic stress. The number of dead cells was based on the measurement of the extracellular activity of released dead-cell protease in coronary effluents, normalized to CF, and served as a level of cytotoxicity in isolated hearts.

### 2.10. Assessment of Oxidative Stress in Rat Hearts

OxiSelect™ In Vitro ROS/RNS Assay Kit (Cell Biolabs, San Diego, USA) was used to document an influence of Klotho on the level of total reactive oxygen and nitrogen species (ROS/RNS) in the rat heart tissue. The assay measures total ROS and RNS, including hydrogen peroxide, nitric oxide, peroxyl radical, and peroxynitrite anion, using a proprietary fluorogenic probe, dichlorodihydrofluorescin DiOxyQ (DCFH-DiOxyQ). The probe is primed with a dequenching reagent to the highly reactive DCFH form. In the presence of ROS and RNS, the DCFH is rapidly oxidized to the highly fluorescent 2′, 7′-dichlorodihydrofluorescein (DCF). Fluorescence intensity is proportional to the total ROS/RNS level within the sample. The total ROS/RNS level was assessed in coronary effluents and normalized to CF.

### 2.11. Measurement of Total Antioxidant Capacity in Rat Hearts

To document an influence of Klotho on oxidative stress resistance during I/R, OxiSelect™ Total Antioxidant Capacity (TAC) Assay Kit (Cell Biolabs, San Diego, USA) was used. Measurement of the total nonenzymatic antioxidant capacity (TAC) is indicative of cells' ability to counteract induced oxidative stress. Briefly, TAC Assay is based on the reduction of copper (II) to copper (I) by the antioxidants present in the sample. Upon reduction, the copper (I) ion further reacts with a coupling chromogenic reagent that produces a color with a maximum absorbance at 490 nm. Absorbance values were proportional to the total reductive capacity in the hearts. TAC level was measured in heart homogenates and normalized to total protein concentration.

### 2.12. Analysis of MLC1 and TnI Concentration in Coronary Effluents

The concentration of ventricular isoform of myosin light chain 1 (MLC1) in perfusates was determined using Rat Myosin Light Chain 3 ELISA Kit (Bioassay Technology Laboratory, Shanghai, China). Briefly, primary capture antibodies bind MLC1 from the sample. Then, MLC1 is detected with biotinylated anti-MLC1 secondary antibodies and streptavidin-horseradish peroxidase (HRP) complex. The substrate solution is then added, and color develops in proportion to the amount of rat MLC1.

Cardiac troponin I (TnI) in coronary effluents was quantitatively measured using Rat Cardiac Troponin I SimpleStep ELISA Kit (Abcam, Cambridge, UK). Briefly, TnI is tied with antibody specific to rat cardiac muscle TnI and is detected by biotin-conjugated polyclonal antibody and HRP. Next, TMB Development Solution is used to enable visualization of the reaction. The concentration of MLC1 and TnI in coronary effluents was normalized to CF.

### 2.13. Determination of Total Protein Concentration

The concentration of total protein in the cardiac tissue and coronary effluents was measured by the Bradford method using Bio-Rad Protein Assay Dye Reagent (Bio-Rad) and Spark multimode microplate reader (Tecan Trading AG, Switzerland). BSA (heat shock fraction, ≥98%, Sigma-Aldrich) served as the protein standard.

### 2.14. Statistical Analysis

Experimental data were analysed using GraphPad Prism 6 software (GraphPad Software, San Diego, CA, USA). The normality of variance changes was calculated with the Shapiro-Wilk normality test or Kolmogorov-Smirnov test. Student's *t*-test or Mann–Whitney *U* tests were used to analyse the data between two groups. The analysis of data in multiple groups was performed with ANOVA or nonparametric test with post hoc tests. Correlations were assessed using Pearson's or Spearman's tests. Results were expressed as mean ± SEM and a value of *p* < 0.05 was regarded as statistically significant.

## 3. Results

### 3.1. An Influence of Klotho Protein on Heart Contractility

**Figure 2:**
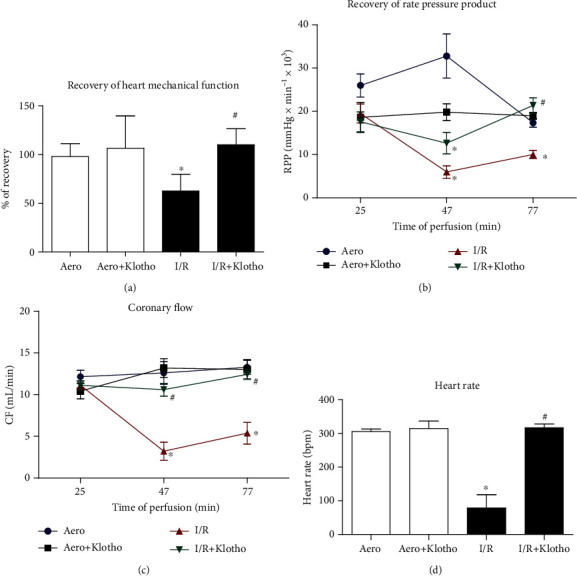
An effect of global I/R injury and Klotho protein on heart contractile function: (a) recovery of heart mechanical function. Percent recovery was calculated as a difference between RPP at 25 and 75 min of perfusion protocol; (b) RPP calculated as the product of the heart rate and pressure developed in the left ventricle (intraventricular pressure of left ventricle × heart rate/1000); (c) coronary flow; (d) heart rate at the end of reperfusion (77 min). Bpm: beats per minute; CF: coronary flow; I/R: ischaemia/reperfusion; RPP: rate pressure product; mean ± SEM; *n*_aero_ = 12; *n*_aero+Klotho_ = 3; *n*_I/R_ = 14; *n*_I/R+Klotho_ = 7; ^∗^*p* < 0.05 vs. Aero; ^#^*p* < 0.05 vs. I/R; ANOVA.

Subjecting the isolated rat hearts to I/R protocol resulted in a significant decrease in cardiac haemodynamic parameters (Table 1, Figure 2). Cardiac mechanical function was decreased approximately by 40% in hearts subjected to I/R in comparison to aerobically perfused hearts (approximately 100% of recovery), showing heart dysfunction (Figure 2(a)). To check the cytotoxicity of Klotho protein, hearts from the aerobic control group were perfused aerobically with Klotho. Supplementation of aerobically perfused hearts with Klotho protein did not affect any of the measured parameters in comparison to the aero group (Table 1, Figure 2). Perfusion of hearts subjected to I/R with Klotho protein resulted in a full recovery of heart contractility (average recovery 110%) (Figure 2(a)) and in a significant increase in cardiac haemodynamic parameters, such as RPP (Table 1, Figure 2(b)), CF (Table 1, Figure 2(c)), HR (Figure 2(d)), and LVDP (Table 1), in comparison to I/R hearts.

**Figure 3:**
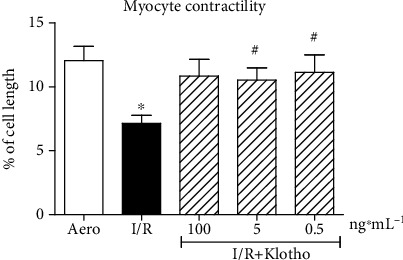
An influence of Klotho protein on isolated rat cardiomyocyte contractility. The contractility was expressed as peak shortening (%) in comparison to the length of the diastolic cell. Mean ± SEM; *n* = 6; ^∗^*p* < 0.05 vs. Aero; ^#^*p* < 0.05 vs. I/R; ANOVA.

An isolated rat cardiomyocyte contractility, expressed as a peak shortening (% of cell length), was significantly reduced in cells subjected to I/R in comparison to group maintained in aerobic condition (Figure 3). Supplementation of the cardiomyocytes with Klotho protein contributed to the maintenance of cell contractility inversely to Klotho dose. Klotho at the concentration of 0.5 ng/mL supported the increase of contractility, whereas 100 ng/mL represented a subthreshold dose that did not significantly maintain contractile function in the cardiomyocytes subjected to I/R (Figure 3).

### 3.2. An Influence of Klotho on the Magnitude of Heart I/R Injury

**Figure 4:**
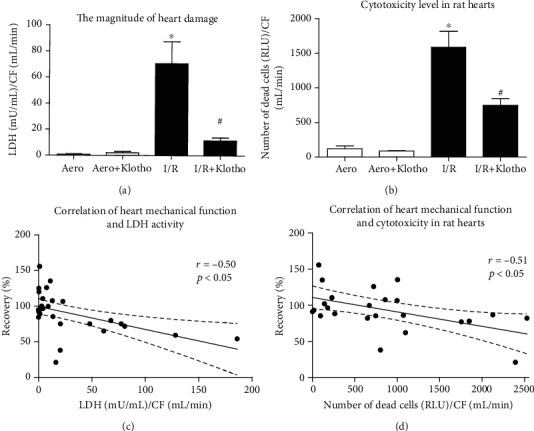
An influence of Klotho on the magnitude of heart I/R injury: (a) the activity of LDH in coronary effluents as a marker of heart injury. LDH activity was normalized to CF; (b) the number of dead cells in rat hearts based on the activity of dead cell protease, tested by cytotoxicity assay. The data was expressed in RLU and normalized to CF; (c) correlation of heart mechanical function and LDH activity; (d) correlation of heart mechanical function and cytotoxicity in rat hearts. CF: coronary flow; LDH: lactate dehydrogenase; mU/mL: milli-international enzyme units per millilitre; RLU: relative light units; mean ± SEM; *n*_aero_ = 9; *n*_aero+Klotho_ = 3; *n*_I/R_ = 10; *n*_I/R+Klotho_ = 7; ^∗^*p* < 0.05 vs. Aero; ^#^*p* < 0.05 vs. I/R; ANOVA.

The measurement of the activity of LDH released from hearts and the number of cardiac dead cells (cytotoxicity level) served as a marker of heart injury. The activity of LDH (Figure 4(a)) and the number of cardiac dead cells (Figure 4(b)) were significantly increased in hearts that underwent I/R procedure compared to the aerobic control group. To check the toxicity of Klotho protein on cardiac tissue, hearts from the aerobic control group were treated with Klotho. Aerobic perfusion with recombinant Klotho protein did not cause cell injury (Figures 4(a) and 4(b)). Administration of Klotho protein to hearts subjected to I/R led to almost full protection against injury (Figure 4(a)) and decreased cytotoxicity (Figure 4(b)) in comparison to the I/R group. Heart mechanical function negatively correlated with LDH activity (*r* = −0.50, *p* < 0.05) (Figure 4(c)) and cytotoxicity level (*r* = −0.51, *p* < 0.05) (Figure 4(d)), showing heart contractile dysfunction due to injury.

### 3.3. Oxidative Status in the Hearts Subjected to I/R Injury

**Figure 5:**
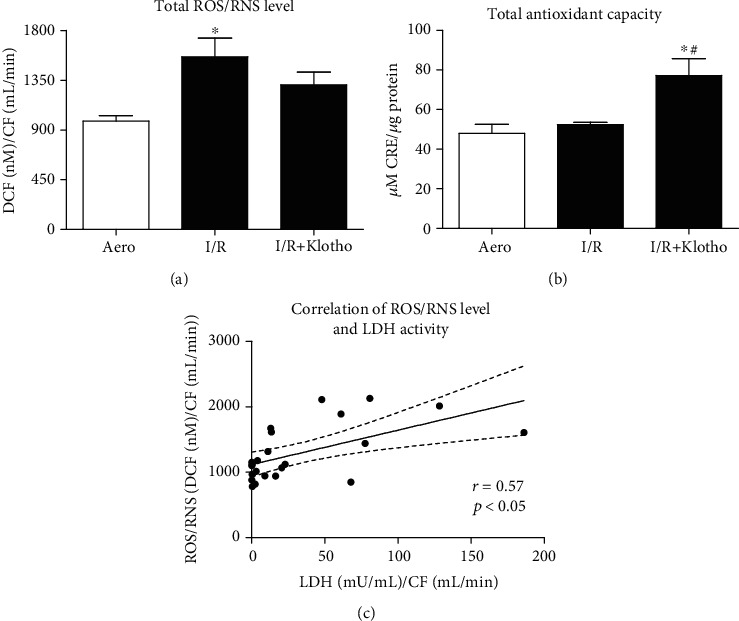
Oxidative status in the hearts subjected to I/R injury: (a) total ROS and RNS level expressed as nM of DCF and normalized to coronary flow; (b) total antioxidant capacity of hearts subjected to I/R. TAC was expressed as *μ*M of CRE and normalized to total protein concentration; (c) correlation of ROS/RNS level and LDH activity. CF: coronary flow; CRE: copper reducing equivalents; DCF: 2′, 7′-dichlorodihydrofluorescein; LDH: lactate dehydrogenase; mU/mL: milli international enzyme units per millilitre; RNS: reactive nitrogen species; ROS: reactive oxygen species; TAC: total antioxidant capacity; mean ± SEM; *n*_aero_ = 8; *n*_I/R_ = 9; *n*_I/R+Klotho_ = 6; ^∗^*p* < 0.05 vs. Aero; ^#^*p* < 0.05 vs. I/R; ANOVA.

Total ROS/RNS level in rat hearts was significantly higher in the I/R group compared to the aerobic control group, confirming oxidative/nitrosative stress (Figure 5(a)). Administration of Klotho protein slightly reduced the production of ROS/RNS (Figure 5(a)) and significantly enhanced total antioxidant capacity (TAC) in hearts subjected to I/R (Figure 5(b)). ROS/RNS level positively correlated with LDH activity (*r* = 0.57, *p* < 0.05) (Figure 5(c)).

### 3.4. Release of Contractile Proteins from Hearts during I/R

**Figure 6:**
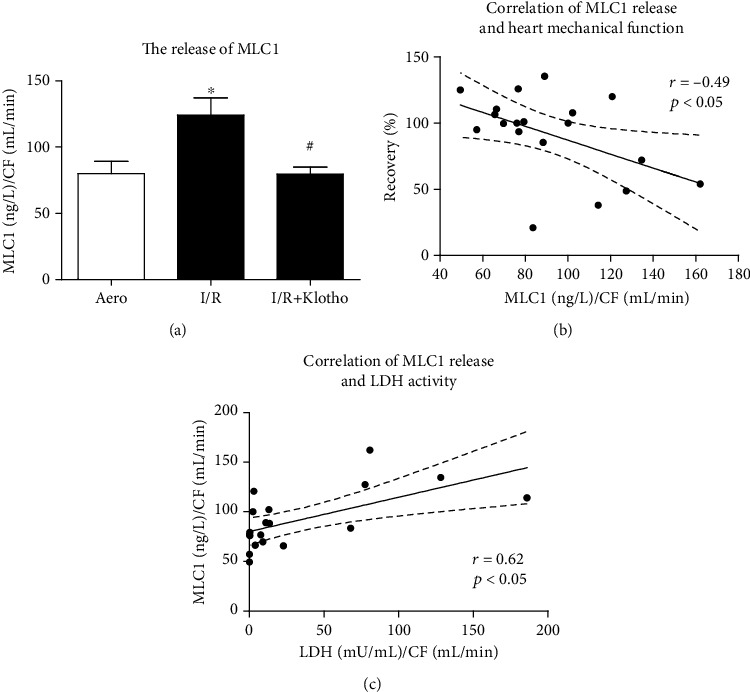
An influence of Klotho protein on MLC1 amount in rat hearts: (a) concentration of MLC1 in coronary effluents tested by ELISA and normalized to CF; (b) correlation of MLC1 concentration and heart mechanical function; (c) correlation of MLC1 concentration and LDH activity. CF: coronary flow; GAPDH: glyceraldehyde 3-phosphate dehydrogenase; LDH: lactate dehydrogenase; mU/mL: milli-international enzyme units per millilitre; MLC1: myosin light chain 1; mean ± SEM; *n*_aero_ = 7; *n*_I/R_ = 5; *n*_I/R+Klotho_ = 7; ^∗^*p* < 0.05 vs. Aero; ^#^*p* < 0.05 vs. I/R; ANOVA.

MLC1 concentration was increased in coronary effluents from hearts subjected to I/R injury compared to aerobic control, showing increased release of MLC1 from the hearts (Figure 6(a)). Administration of Klotho protein to I/R hearts effectively reduced the release of MLC1 (Figure 6(a)). The concentration of MLC1 in coronary effluents negatively correlated with heart mechanical function (*p* < 0.05, *r* = −0.49) (Figure 6(b)) and positively correlated with LDH activity (*p* < 0.05, *r* = 0.62) (Figure 6(c)), confirming the mechanism of I/R injury.

**Figure 7:**
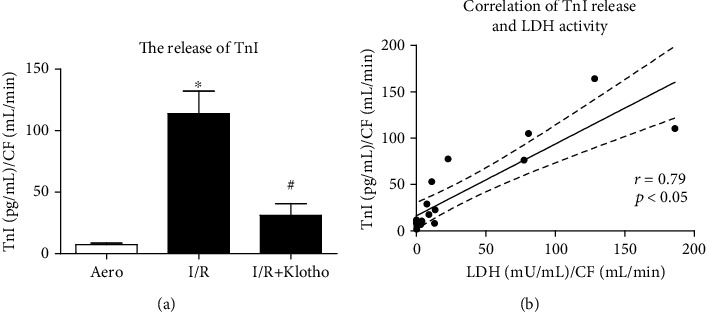
An influence of Klotho protein on TnI release in rat hearts: (a) concentration of TnI in coronary effluents tested by ELISA and normalized to CF; (b) correlation of TnI concentration and LDH activity. CF: coronary flow; LDH: lactate dehydrogenase; mU/mL: milli-international enzyme units per millilitre; TnI: troponin I; mean ± SEM; *n*_aero_ = 7; *n*_I/R_ = 5; *n*_I/R+Klotho_ = 7; ^∗^*p* < 0.05 vs. Aero; ^#^p < 0.05 vs. I/R; ANOVA.

The content of TnI in coronary effluents from hearts subjected to I/R injury was increased compared to aerobic control (Figure 7(a)). Administration of Klotho protein during I/R contributed to reduced release of TnI (Figure 7(a)). The release of TnI positively correlated with LDH activity (*r* = 0.79, *p* < 0.05) (Figure 7(b)).

## 4. Discussion

Kidneys are considered to be the major site of Klotho expression and primary source of the circulating protein. However, Klotho is also produced in other types of tissue, like the skeletal muscle, adipose tissues, brain, or liver [5, 8]. Recent studies have underlined the importance of Klotho in the cardiac muscle too [16]. We had previously reported compensative production of Klotho in the rat hearts to protect cardiac tissue from I/R injury. Enhanced production and release of Klotho into the extracellular space were also proposed as a biomarker of heart damage [12]. Therefore, the main aim of this investigation was to examine the influence of Klotho administration on the rat hearts injured by ischaemia/reperfusion. Our research showed that Klotho protein contributed to the reduction of damage and contractile dysfunction in the heart during I/R. We strongly suggest that Klotho can be valuable as a potential agent that supports cardioprevention/protection in ischaemic heart disorders.

In this study, subjecting the isolated rat hearts to the I/R procedure resulted in a significant decrease in haemodynamic parameters. The administration of Klotho protein during I/R supported the recovery of heart mechanical function and contractility of isolated cardiomyocytes. Our results correspond to the research of Hui et al., where treatment with recombinant Klotho after endotoxemia-induced heart injury improved the haemodynamic parameters and cardiac function in aging mice [17]. It is known that ROS directly influence contractile function by modifying proteins involved in excitation-contraction coupling. The suppression of calcium channels and oxidative interaction with Ca^2+^ ATPase in the sarcoplasmic reticulum occur. It results in the inhibition of Ca^2+^ uptake. Thus, intracellular and mitochondrial Ca^2+^ overload contributes to cardiac contractile disorders [3, 18]. It was found that membrane-bound Klotho is a coreceptor for fibroblast growth factor 23 (FGF23) and plays a role in ion homeostasis. Soluble Klotho can induce negative phosphate and calcium balance also independently of FGF23 as an endocrine, autocrine, and paracrine hormone [6, 19]. Importantly, stress-induced heart hypertrophy and remodelling were impaired by means of Klotho. Soluble Klotho was recognized as an inhibitor of TRPC6 channel exocytosis in the cardiomyocytes. Thus, Klotho protected the heart during stress conditions by downregulating of Ca^2+^ influx and abnormal Ca^2+^ signalling [6, 20–22]. It was also shown that Klotho acted as an endogenous inhibitor of calcification and mediated between FGF23 and vascular cells [23]. The current research showed that Klotho supported contractility in the injured cardiomyocytes. Taking into account that Klotho influences calcium homeostasis in the heart, it may contribute to the prevention of cardiac mechanical function and contractile disorders. However, further studies are needed to explore the underlying molecular mechanism of the potential effect of Klotho on calcium-dependent heart contractility.

There are studies showing that Klotho protein has been characterized as a factor in the prevention of oxidative stress, inflammation, fibrosis, and apoptosis [7, 9, 10, 24]. Moreover, Klotho deficiency has been identified in many disorders such as inflammatory bowel disease, kidney disease, atherosclerosis, cardiac hypertrophy, and remodelling [10, 16, 20, 25–27]. In our previous research, human cardiomyocytes subjected to I/R injury produced Klotho in a compensative manner to protect from the damage [12]. In the present study, subjecting the rat hearts to I/R caused injury and increased production of ROS/RNS in the cardiac tissue. Heart mechanical function negatively correlated with the markers of injury, showing heart contractile dysfunction due to I/R. There was also a positive correlation of ROS/RNS level and LDH activity, indicating the heart damage due to overproduction of ROS/RNS. Klotho protein contributed to the reduction of cardiac injury and cytotoxicity and supported the production of antioxidants and antioxidant capacity in I/R hearts. However, only a slight decline of ROS/RNS level in the I/R+Klotho group compared to the I/R group does not explain the protective mechanism of Klotho. Further study is needed to clarify this concatenation. Moreover, the magnitude of damage and the number of dead cells were significantly lower after supplementation of the cardiac muscle with Klotho. These data were confirmed in other studies showing that Klotho prevents cell apoptosis and cytotoxic activity after induced oxidative stress in other organs [9, 11, 27–29]. Klotho also reduced the apoptosis in an animal model of I/R kidney injury or in a mouse kidney cell line after exposure to hydrogen peroxide [10, 27, 30]. Similarly, the level of necroptotic markers after renal I/R injury in mice was ameliorated by Klotho due to oxidative stress inhibition [31]. There was also a correlation of the neuroprotection and inhibition of neuropathological changes with the induced expression of Klotho after ischaemic injury in the murine brain [11]. Finally, the apoptosis and heart damage caused by endotoxemia or hyperglycaemia in mice were alleviated by Klotho [17, 32]. Our previous research revealed improved viability and metabolic activity, as well as reduced injury after Klotho administration in human cardiomyocytes subjected to I/R [12]. The current study showed the potential influence of Klotho on cellular defence against oxidative stress, reduction of cell death, and injury at the tissue level in I/R rat hearts. As previously mentioned, Klotho protein was characterized by its antioxidative activity. Klotho had a distinct capacity to inhibit the production of ROS [9, 11, 29, 31–34]. Importantly, it was revealed that Klotho led to enhanced expression of oxidative scavengers like superoxide dismutase (SOD) and antioxidation through the inhibition of phosphatidyl inositol-3 kinase (PI3K)/protein kinase B (Akt) signalling pathway [9, 28, 29, 34]. Thus, Klotho may contribute to the promotion of antioxidation and protection against apoptosis during heart I/R.

Based on present knowledge, the main contributors to I/R injury of the heart are increased production of ROS, enhanced expression of nitric oxide synthase (NOS), overproduction of nitric oxide (NO), subsequent activation of MMPs, and proteolytic degradation of cardiac contractile proteins [3, 35–39]. It was found that ROS and RNS contribute to nitration/nitrosylation of heart contractile proteins and activate MMPs, including MMP-2 and MMP-9 [37–39]. It was shown that MMP-2 not only acts in the extracellular matrix but also functions at the cellular level in the cardiomyocytes [40]. Heart contractile proteins like myosin light chains 1 (MLC1) or troponin are nitrated, nitrosylated, and phosphorylated during I/R, which increases their affinity for the proteolytic enzymes. As a result, MLC1 is then degraded by MMPs. It was confirmed that degradation of MLC1 during I/R injury leads to contractile dysfunction in the cardiac muscle [38, 41]. It was reported that Klotho contributed to decrease in iNOS expression in human endothelial cells during oxidative stress. The expression of MMP-2 and MMP-9 in the kidneys was also reduced by means of Klotho [24, 33, 42–44]. In the present research, the level of MLC1 and TnI in coronary effluents was increased after I/R injury of the rat hearts. The release of MLC1 and TnI positively correlated with the magnitude of heart damage and negatively correlated with heart mechanical function, confirming the mechanism of I/R injury. Importantly, we have previously reported that the administration of inhibitors of NOS, MMP-2, and MLC phosphorylation resulted in cardioprotection of the human cardiomyocytes or isolated rat hearts [13–15, 45, 46]. The current study showed reduced release of MLC1 and TnI during I/R after Klotho administration. For this reason, we hypothesize that Klotho may contribute to the protection of heart contractility throughout limitation of oxidative/nitrosative stress and subsequent reduction of MMP-dependent degradation of MLCs. Thus, the next goal of our investigation is to elucidate the underlying mechanism of Klotho influence in relation to ROS/RNS and MLC1. The analysis of NOS, NO, MMP-2, MMP-9, and tissue inhibitors of metalloproteinases levels will be then performed.

## 5. Conclusion

In conclusion, the presented research showed that the administration of Klotho protein to the heart during I/R injury enhanced antioxidant capacity and supported the reduction of cell death, toxicity, and release of heart contractile proteins. We are also aware that confirmation of the potential influence and cardioprotection of Klotho in the heart needs further studies.

## Figures and Tables

**Table 1:** An influence of Klotho on cardiac mechanical function of isolated rat hearts.

Parameter	Aero	Aero+Klotho	I/R	I/R+Klotho
dP/dt_max_ (mmHg/s)^†^	1559 ± 93	1407 ± 72	1227 ± 57^∗^	1494 ± 157
PED (mmHg)^†^	9.9 ± 0.9	10.6 ± 0.8	8.6 ± 0.5^∗^	9.1 ± 0.2
LVDP (mmHg)^†^	57.3 ± 2.4	61.0 ± 4.9	40.5 ± 4.8^∗^	66.5 ± 6.2^#^
RPP (mmHg × min^−1^ × 10^3^)^†^	17.8 ± 1.0	19.0 ± 0.8	9.9 ± 1.1^∗^	21.4 ± 1.8^#^
CF (mL/min)^‡^	12.5 ± 1.6	13.2 ± 1.1	3.4 ± 1.1^∗^	10.6 ± 0.8^#^

CF: coronary flow; dP/dt_max_: baseline left ventricular maximal contractility; I/R: ischaemia/reperfusion; LVDP: left ventricular developed pressure; PED: left ventricular end-diastolic pressure; RPP: rate pressure product; mean ± SEM; *n*_aero_ = 12; *n*_aero+Klotho_ = 3; *n*_I/R_ = 14; *n*_I/R+Klotho_ = 7; ^∗^*p* < 0.05 vs. Aero; ^#^*p* < 0.05 vs. I/R; ANOVA. ^†^After I/R (77 min of the experiment); ^‡^after ischaemia (first minute of reperfusion).

## Data Availability

The data that support the findings of this study are available from the corresponding author upon reasonable request.

## References

[1] Antman E. M., Anbe D. T., Armstrong P. W. (2004). ACC/AHA guidelines for the management of matients with ST-elevation myocardial infarction—executive summary: a report of the American College of Cardiology/American Heart Association Task Force on Practice Guidelines (writing committee to revise the 1999 guidelines for the management of patients with acute myocardial infarction). *Circulation*.

[2] Heusch G., Gersh B. J. (2017). The pathophysiology of acute myocardial infarction and strategies of protection beyond reperfusion: a continual challenge. *European Heart Journal*.

[3] Tsutsui H., Kinugawa S., Matsushima S. (2011). Oxidative stress and heart failure. *American Journal of Physiology. Heart and Circulatory Physiology*.

[4] Kuro-o M. (2006). Klotho as a regulator of fibroblast growth factor signaling and phosphate/calcium metabolism. *Current Opinion in Nephrology and Hypertension*.

[5] Kuro-o M., Matsumura Y., Aizawa H. (1997). Mutation of the mouse klotho gene leads to a syndrome resembling ageing. *Nature*.

[6] Kim J.-H., Hwang K.-H., Park K.-S., Kong I. D., Cha S. K. (2015). Biological role of anti-aging protein Klotho. *Journal of Lifestyle Medicine*.

[7] Olejnik A., Franczak A., Krzywonos-Zawadzka A., Kałużna-Oleksy M., Bil-Lula I. (2018). The biological role of Klotho protein in the development of cardiovascular diseases. *BioMed Research International*.

[8] Markan K. R., Naber M. C., Small S. M., Peltekian L., Kessler R. L., Potthoff M. J. (2017). FGF21 resistance is not mediated by downregulation of beta-klotho expression in white adipose tissue. *Molecular Metabolism*.

[9] Yamamoto M., Clark J. D., Pastor J. V. (2005). Regulation of oxidative stress by the anti-aging hormone klotho. *The Journal of Biological Chemistry*.

[10] Sugiura H., Yoshida T., Tsuchiya K. (2005). Klotho reduces apoptosis in experimental ischaemic acute renal failure. *Nephrology, Dialysis, Transplantation*.

[11] Zhou H.-J., Li H., Shi M.-Q. (2018). Protective effect of Klotho against ischemic brain injury is associated with inhibition of RIG-I/NF-*κ*B signaling. *Frontiers in Pharmacology*.

[12] Olejnik A., Krzywonos-Zawadzka A., Banaszkiewicz M., Bil-Lula I. (2020). Klotho protein contributes to cardioprotection during ischaemia/reperfusion injury. *Journal of Cellular and Molecular Medicine*.

[13] Krzywonos-Zawadzka A., Franczak A., Olejnik A. (2019). Cardioprotective effect of MMP-2-inhibitor-NO-donor hybrid against ischaemia/reperfusion injury. *Journal of Cellular and Molecular Medicine*.

[14] Krzywonos-Zawadzka A., Wozniak M., Sawicki G., Bil-Lula I. (2020). A drug cocktail for protecting against ischemia-reperfusion injury. *Frontiers in Bioscience*.

[15] Bil-Lula I., Lin H.-B., Biały D. (2016). Subthreshold nitric oxide synthase inhibition improves synergistic effects of subthreshold MMP-2/MLCK-mediated cardiomyocyte protection from hypoxic injury. *Journal of Cellular and Molecular Medicine*.

[16] Corsetti G., Pasini E., Scarabelli T. M. (2016). Decreased expression of Klotho in cardiac atria biopsy samples from patients at higher risk of atherosclerotic cardiovascular disease. *Journal of Geriatric Cardiology*.

[17] Hui H., Zhai Y., Ao L. (2017). Klotho suppresses the inflammatory responses and ameliorates cardiac dysfunction in aging endotoxemic mice. *Oncotarget*.

[18] Marks A. R. (2003). Calcium and the heart: a question of life and death. *The Journal of Clinical Investigation*.

[19] Kurosu H., Ogawa Y., Miyoshi M. (2006). Regulation of fibroblast growth factor-23 signaling by klotho. *The Journal of Biological Chemistry*.

[20] Xie J., Cha S.-K., An S.-W., Kuro-o M., Birnbaumer L., Huang C.-L. (2012). Cardioprotection by Klotho through downregulation of TRPC6 channels in the mouse heart. *Nature Communications*.

[21] Dalton G. D., Xie J., An S.-W., Huang C. L. (2017). New insights into the mechanism of action of soluble klotho. *Frontiers in Endocrinology*.

[22] Leunissen E. H. P., Nair A. V., Büll C. (2013). The epithelial calcium channel TRPV5 is regulated differentially by klotho and sialidase. *The Journal of Biological Chemistry*.

[23] Lim K., Lu T.-S., Molostvov G. (2012). Vascular Klotho deficiency potentiates the development of human artery calcification and mediates resistance to fibroblast growth factor 23. *Circulation*.

[24] Doi S., Zou Y., Togao O. (2011). Klotho inhibits transforming growth factor-*β*1 (TGF-*β*1) signaling and suppresses renal fibrosis and cancer metastasis in mice. *The Journal of Biological Chemistry*.

[25] Semba R. D., Cappola A. R., Sun K. (2011). Plasma klotho and cardiovascular disease in adults. *Journal of the American Geriatrics Society*.

[26] Matsumura Y., Aizawa H., Shiraki-Iida T., Nagai R., Kuro-o M., Nabeshima Y. I. (1998). Identification of the human Klotho gene and its two transcripts encoding membrane and secreted Klotho protein. *Biochemical and Biophysical Research Communications*.

[27] Mitobe M., Yoshida T., Sugiura H., Shirota S., Tsuchiya K., Nihei H. (2005). Oxidative stress decreases klotho expression in a mouse kidney cell line. *Nephron Experimental Nephrology*.

[28] Lim S. W., Jin L., Luo K. (2017). Klotho enhances FoxO3-mediated manganese superoxide dismutase expression by negatively regulating PI3K/AKT pathway during tacrolimus-induced oxidative stress. *Cell Death & Disease*.

[29] Yao Y., Wang Y., Zhang Y., Liu C. (2017). Klotho ameliorates oxidized low density lipoprotein (ox-LDL)-induced oxidative stress via regulating LOX-1 and PI3K/Akt/eNOS pathways. *Lipids in Health and Disease*.

[30] Sugiura H., Yoshida T., Mitobe M. (2009). Klotho reduces apoptosis in experimental ischaemic acute kidney injury via HSP-70. *Nephrology, Dialysis, Transplantation*.

[31] Qian Y., Guo X., Che L. (2018). Klotho reduces necroptosis by targeting oxidative stress involved in renal ischemic-reperfusion injury. *Cellular Physiology and Biochemistry*.

[32] Guo Y., Zhuang X., Huang Z. (2018). Klotho protects the heart from hyperglycemia-induced injury by inactivating ROS and NF-*κ*B-mediated inflammation both in vitro and in vivo. *Biochimica et Biophysica Acta - Molecular Basis of Disease*.

[33] Richter B., Haller J., Haffner D., Leifheit-Nestler M. (2016). Klotho modulates FGF23-mediated NO synthesis and oxidative stress in human coronary artery endothelial cells. *Pflügers Archiv*.

[34] Takenaka T., Kobori H., Inoue T. (2017). [op.4b.02] klotho supplementation attenuates blood pressure and oxidative stress in diabetes. *Journal of Hypertension*.

[35] Byrne J. A., Grieve D. J., Cave A. C., Shah A. M. (2003). Oxidative stress and heart failure. *Archives des Maladies du Coeur et des Vaisseaux*.

[36] Marfella R., Di Filippo C., Esposito K. (2004). Absence of inducible nitric oxide synthase reduces myocardial damage during ischemia reperfusion in streptozotocin-induced hyperglycemic mice. *Diabetes*.

[37] Wang W., Schulze C. J., Suarez-Pinzon W. L., Dyck J. R. B., Sawicki G., Schulz R. (2002). Intracellular action of matrix metalloproteinase-2 accounts for acute myocardial ischemia and reperfusion Injury. *Circulation*.

[38] Sawicki G., Leon H., Sawicka J. (2005). Degradation of myosin light chain in isolated rat hearts subjected to ischemia-reperfusion injury. *Circulation*.

[39] Wang W., Sawicki G., Schulz R. (2002). Peroxynitrite-induced myocardial injury is mediated through matrix metalloproteinase-2. *Cardiovascular Research*.

[40] Kwan J. A., Schulze C. J., Wang W. (2004). Matrix metalloproteinase-2 (MMP-2) is present in the nucleus of cardiac myocytes and is capable of cleaving poly (ADP-ribose) polymerase (PARP) in vitro. *The FASEB Journal*.

[41] Cadete V. J. J., Sawicka J., Jaswal J. S. (2012). Ischemia/reperfusion-induced myosin light chain 1 phosphorylation increases its degradation by matrix metalloproteinase 2. *FEBS Journal*.

[42] Chang B., Kim J., Jeong D. (2012). Klotho inhibits the capacity of cell migration and invasion in cervical cancer. *Oncology Reports*.

[43] Wu Y.-L., Xie J., An S.-W. (2017). Inhibition of TRPC6 channels ameliorates renal fibrosis and contributes to renal protection by soluble klotho. *Kidney International*.

[44] Cheng X., Zhou Q., Lin S., Wu R. (2010). Fosinopril and valsartan intervention in gene expression of Klotho, MMP-9, TIMP-1, and PAI-1 in the kidney of spontaneously hypertensive rats. *Zhong Nan Da Xue Xue Bao. Yi Xue Ban*.

[45] Krzywonos-Zawadzka A., Franczak A., Sawicki G., Bil-Lula I. (2020). Mixture of MMP-2, MLC, and NOS inhibitors affects NO metabolism and protects heart from cardiac I/R injury. *Cardiology Research and Practice*.

[46] Bil-Lula I., Krzywonos-Zawadzka A., Sawicka J. (2018). L-NAME improves doxycycline and ML-7 cardioprotection from oxidative stress. *Frontiers in Bioscience*.

