# Corrigendum to “Ameliorating Effect of Klotho Protein on Rat Heart during I/R Injury”

**DOI:** 10.1155/2022/9781367

**Published:** 2022-07-04

**Authors:** Agnieszka Olejnik, Anna Krzywonos-Zawadzka, Marta Banaszkiewicz, Iwona Bil-Lula

**Affiliations:** Division of Clinical Chemistry and Laboratory Hematology, Department of Medical Laboratory Diagnostics, Faculty of Pharmacy, Wroclaw Medical University, Borowska 211A St., 50-556 Wroclaw, Poland

In the article titled “Ameliorating Effect of Klotho Protein on Rat Heart during I/R Injury” [1], Acknowledgments should read as follows.
